# A sequential model of two-choice intensity identification

**DOI:** 10.3389/fcogn.2025.1561842

**Published:** 2025-03-19

**Authors:** Robert C. G. Johansson, Rolf Ulrich

**Affiliations:** Eberhard Karls Universität Tübingen, Tübingen, Germany

**Keywords:** intensity identification, Poisson model, sequential testing, stochastic evidence accumulation, perceptual decision-making

## Abstract

A model of perceptual decision-making in two-choice intensity identification tasks is advanced. The model assumes that sensory pathways encode the physical intensity of the stimulus in the firing rates of sensory afferents, characterized by exponentially distributed interarrival times. The decision-making process entails a sequential comparison of each interarrival time with memory traces from prior stimulus exposure. This yields a random walk process reminiscent of the two-choice RT model by Stone (1960), but with an additional stochastic element introduced by variable sampling times. The model provides a reasonable account of data garnered in a brightness identification task (Experiment 1), aligning with distributional RT statistics and intensity effects on mean RTs. Several *post hoc* assumptions, such as variability and bias in the starting point of the random walk, are required to accurately predict error RT distributions, however, which introduces problematic asymmetries in predicted error probabilities. Applying the model to a loudness identification task (Experiment 2) necessitated the additional assumption of variability in transduction rates to overcome challenges in accommodating longer RTs for errors compared to correct responses in this task.

## Introduction

The ability to discern events of differing physical intensity is a foundational cognitive ability. Therefore, researchers in experimental psychology have long sought to unravel how the physical intensity of the stimulus translates into our internal realm of sensation (Fechner, 1860; Catell, 1886; Stevens, 1957; Piéron, 1920; Treisman, 1964). This research sought to investigate our capacity to identify stimuli based on their intensity level, as operationalized in cognitive tasks relying on the method of absolute intensity judgments (Wever and Zener, 1928; Warden and Rowley, 1929; Wolfle, 1937). In the absolute intensity judgment paradigm, participants are presented with a stimulus such as a light or a tone. This stimulus is varied along a unidimensional intensity continuum on a trial-by-trial basis, taking on one of several predefined intensity levels. The task of the observer is, then, to determine the identity of the presented stimulus with respect to its luminance level or sound pressure level, for example. Because there is a direct, one-to-one mapping between stimulus value and response category in this task, it is also commonly referred to as the *identification task*.

Notably, the intricacy of behavioral measurement in identification paradigms escalates rapidly with increasing stimulus set size. For a set of *n* intensity levels, each stimulus can conceivably be confused with *n*−1 other stimuli, or be correctly identified, so the number of response probabilities to be estimated in this task is generally *n*^2^. The measurement of reaction times (RTs) in identification tasks poses similar problems when stimulus set is large; in particular, when distributional analyses of incorrect RTs are warranted but confusion probabilities are small. Because rigorous treatment of RT statistics was imperative for this research, empirical and theoretical efforts were channeled toward the two-choice identification task wherein the stimulus can take on only two possible intensity levels. Nonetheless, a brief introduction to the broader topic of absolute intensity judgments is provided below for context and perspective.

### Brief sketch of intensity identification

At best, people can correctly identify perhaps a handful of intensity levels in the identification task, with rapid deterioration of performance as stimulus set grows large (as reviewed in the famous magic number 7 ± 2 paper of Miller, 1956). This finding holds true for every single sensory modality and intensity domain studied, including visual brightness (Garner, 1953; Landau et al., 1974; Holland and Lockhead, 1968), auditory loudness (Landau et al., 1974; Eriksen, 1953; Luce et al., 1976; Nosofsky, 1983a; Luce and Nosofsky, 1984; Sagi et al., 2007), heaviness of weights (Wever and Zener, 1928), amplitude of cutaneous electric current (Hawkes and Warm, 1960), and the chemical concentration of taste stimuli and odorants (Beebe-Center et al., 1955; Engen and Pfaffmann, 1959). This limitation stands in stark contrast to people's remarkable ability to discriminate between a much larger set of intensity levels—potentially numbering in the hundreds—in the context of comparative judgment paradigms. Expanding the dynamic range of the stimulus set does not alleviate this state of affairs (Pollack, 1952; Alluisi, 1957; Luce et al., 1976; Luce and Nosofsky, 1984), hinting that this limitation cannot be solely attributed to poor intensity resolution in sensory pathways. Even non-human species of animals such as rats and pigeons seem to struggle with absolute judgments of intensity, usually exhibiting very gradually inclining learning curves in this task, although the same animal might provide comparative judgments in paired discrimination paradigms with relative ease (Warden and Rowley, 1929; Wolfle, 1937).

In the early investigations of absolute judgments, response probability emerged as the primary dependent variable of theoretical interest (Garner, 1953; Miller, 1956; Alluisi, 1957; Eriksen, 1953; Pollack, 1952) whereas RTs received limited attention with only a few exceptions (e.g., Doherty, 1968; Bevan and Avant, 1968; Pachella and Fisher, 1972). This historical neglect of response latency reverberates through numerous attempts to model the cognitive mechanisms underlying performance in intensity identification. Notable examples include the trace-context model (Durlach and Braida, 1969; Braida and Durlach, 1972), the attention band model (Luce et al., 1976; Nosofsky, 1983b), and the shifting categories model (Purks et al., 1980). These Thurstonian models, firmly rooted in the theory of signal detection (Green and Swets, 1966), are ultimately static frameworks and do not offer a comprehensive account of the temporal dynamics of perceptual decision-making in this type of task. Perhaps, therefore, studies evaluating signal detection models of identification performance have yielded mixed results, with no clear-cut evidence favoring one theory over the other (Luce and Nosofsky, 1984; Purks et al., 1980; Luce et al., 1982).

Recognizing this gap, modern approaches have underscored the theoretical necessity of a chronometric analysis of identification performance. Notable among these approaches are the connectionist model by Lacouture and Marley (1991), the exemplar-based theories advocated by Nosofsky (1997) and Kent and Lamberts (2005), as well as certain hybrid frameworks incorporating linear ballistic accumulation (Brown et al., 2008) and leaky, competing accumulation (Lacouture and Marley, 2004). A particularly lucid review of much of this work is to be found in Stewart et al. (2005). For our present purposes, it suffices to highlight a single, ubiquitous feature of these RT models, namely: the generality of their scope. More concretely, these models aim to account for absolute judgments in the broadest sense of the term, without specific consideration for the sensory attribute under examination. Implicit in this approach is the assumption that judgments of visual line length, acoustic frequency, or stimulus duration, for example, are mostly interchangeable. It, therefore, seems worthwhile to seek a more psychophysically principled account of absolute intensity judgments that explicitly engages with the representational format of stimulus intensity in sensory pathways. Such an approach may sacrifice some universality, but holds promise to offer a more physiologically plausible framework for understanding absolute intensity judgments.

The remainder of this paper seeks to advance such a framework. Our model bridges two important concepts from the psychophysical information processing literature which have not been linked previously: First, the Poisson approximation approach to sensory encoding (Link, 1992; Luce and Green, 1972; Hildreth, 1979), and second, notions of statistically optimal decision-making as embodied in the Sequential Likelihood Ratio Test (SLRT; Wald, 1945, 1947). These two components form the bedrock of a cognitive process model which seeks to predict both choice RT and response probability in intensity identification tasks. We introduce the proposed Poisson-SLRT model in the context of two-choice paradigms where the stimulus set consists of *n* = 2 signal intensities. We then put it to empirical test in two separate studies examining speeded absolute judgments of brightness level (Experiment 1) and loudness level (Experiment 2).

### Poisson transduction in sensory pathways

The Poisson approximation approach to sensory encoding has been a pivotal element in psychophysical theories of intensity processing (e.g., Luce and Green, 1972; Hildreth, 1979; Link, 1992; Teich et al., 1978; Lachs and Teich, 1981; Treisman, 1966; Hecht et al., 1942) as well as in the domain of time perception (Ulrich et al., 2022; Creelman, 1962; Gibbon, 1992). It departs from the notion that the output of peripheral sensory transducers comprises a stream of neural pulses traveling toward a task-dependent decision mechanism localized somewhere centrally in the brain. How this pulse train unfolds in time is assumed to be fairly approximated by a stationary Poisson process.

The Poisson process, denoted *N*(*t*), is a counting process which describes the number of events (neural pulses in this context) occurring within the time interval *t*. A single impulse occurs within a tiny time interval Δ*t* with probability *p*≈Δ*t*·λ, where λ denotes the rate of the process. The process initiates at stimulus onset and higher intensity levels are associated with a faster rate of neural transmission as embodied in a larger value of λ. When a signal is presented over an interval of length *t*, the probability that *N*(*t*) = *k* pulses arrive at the decision locus within this interval adheres to Poisson's probability law


(1) $$\begin{array}{l} P\left[N\left(t\right)=k\right]=\frac{{\left(\lambda t\right)}^{k}e^{-\lambda t}}{k!}, \end{array}$$

where *k*∈ℕ_0_ is the number of neural events. The quantal nature of these events can be seen to mirror the all-or-nothing characteristic of neuronal transmission, whereby a pulse either occurs or does not occur at any given moment. Importantly, the Poisson process implies that the *interarrival time* (IAT) between two successive pulses is exponentially distributed with mean interpulse time θ = 1/λ. In other words, a faster rate of neural transmission implies a shorter interpulse time.

### Sequential stimulus identification

Imagine an observer tasked with identifying the intensity of a presented stimulus *S*_*i*_ as one of two possible levels, here denoted *i* (with *i* = *w, s*). For example, assume a “weak” signal *S*_*w*_, and a “strong” signal *S*_*s*_, of equal duration. Upon presentation, each signal is then associated with a renewal process which is fully characterized by its respective Poisson rate parameter (λ_*w*_ or λ_*s*_). Again, the stronger signal is associated with a faster rate of neural activation, hence λ_*s*_>λ_*w*_. Necessarily, the observer is given the opportunity to become familiar with the two stimuli through a series of practice trials. We reason that this practice allows the observer to form two faithful memory representations, denoted θ_*w*_ and θ_*s*_, reflecting the mean average IAT generated upon presentation of the corresponding signal intensity. These memory traces are presumed to be sustained throughout the experiment on the observers accord, enabling them to compare incoming sensory information against prior experience.

As mentioned above, the Poisson process implies that the IATs between two neural events at times *T*_*j*−1_ and *T*_*j*_ are exponentially distributed, such that *X*_*j*_ = *T*_*j*_−*T*_*j*−1_ has the expected value *E*[*X*|*S*_*i*_] = 1/λ_*i*_ = θ_*i*_. For each IAT recorded at the decision center, we propose that the observer adjust their beliefs about the state of the world in accordance with the statistically optimal decision rule embodied by the SLRT (Wald, 1947). This implies that for each neural event, the observer internally computes the log-likelihood ratio


(2) $$\begin{array}{lll} \Lambda_{j} & = & \log\left[\frac{L\left(\theta_{s}|X_{j}\right)}{L\left(\theta_{w}|X_{j}\right)}\right] \end{array}$$

(3) $$\begin{array}{ll} = & \log\left[\frac{\frac{1}{\theta_{s}}\exp\left(-X_{j}/\theta_{s}\right)}{\frac{1}{\theta_{w}}\exp\left(-X_{j}/\theta_{w}\right)}\right] \end{array}$$

(4) $$\begin{array}{ll} = & \frac{\theta_{s}-\theta_{w}}{\theta_{s}\theta_{w}}\cdot X_{j}-\log\left(\frac{\theta_{s}}{\theta_{w}}\right). \end{array}$$

As additional pulses reach the decision center, the observer continues to update their beliefs by accumulating further evidence in favor of the two hypotheses. This updating process is described as follows


(5) $$\begin{array}{l} \Sigma_{j}=\Sigma_{j-1}+\Lambda_{j} \end{array}$$

**Figure 1:**
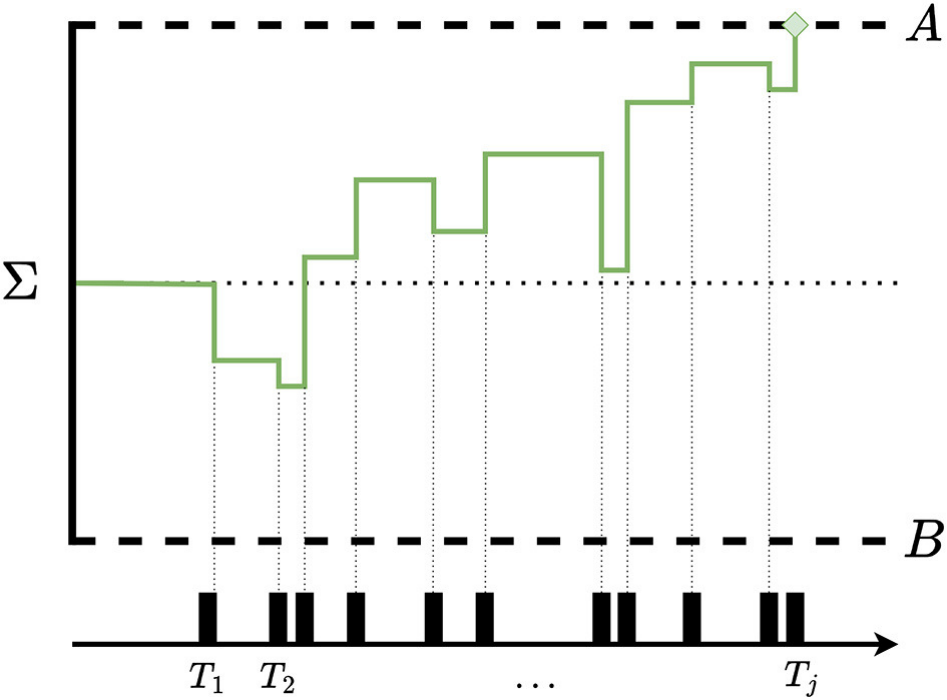
Schematic illustration of the Poisson-SLRT model of two-choice intensity identification. Please note that whenever IATs between successive neural pulses are long, the process is attracted toward the lower barrier *B*. Conversely, the process moves toward the upper barrier *A* when IATs are short.

It can be seen that as sensory evidence begins to gather, Σ enters a random walk over the line of real numbers. This step function is discontinuous at each *T*_*j*_, as schematically illustrated in Figure 1 with the starting point set to Σ_0_ = 0.

The updating process continues for as long as *A*>Σ_*j*_>*B*, where *A* and *B* denote the absorption barriers of the process. As demonstrated by Wald (1945), one can choose *A* and *B* on the basis of the desired Type I (α) and Type II (β) error rates of the procedure such that


(6) $$\begin{array}{l} A\approx \log\frac{\beta }{1-\alpha } \end{array}$$

and


(7) $$\begin{array}{l} B\approx \log\frac{1-\beta }{\alpha }. \end{array}$$

Consequently, the duration *D* to arrive at a decision regarding whether *S*_*w*_ or *S*_*s*_ has been presented is given by


(8) $$\begin{array}{l} D=\min\left(T_{j}:\Sigma_{j}>A\text{ or}\Sigma_{j}<B\right), \end{array}$$

meaning that the updating process terminates as soon as Σ_*j*_ exceeds *A* (evidence for *S*_*s*_) or falls below *B* (evidence for *S*_*w*_). The barriers *A* and *B* can be computed directly from the observed frequencies of decision errors, because of the confusion probabilities α = *P*(*R*_*w*_|*S*_*s*_) and β = *P*(*R*_*s*_|*S*_*w*_), where *R*_*w*_ and *R*_*s*_ denote the responses in favor of *S*_*w*_ and *S*_*s*_, respectively. The total time required for a response to occur is *RT* = *D*+*t*_0_, where *t*_0_ represents a residual component time for non-decision processes such as transmission and motor latencies in the nervous system. Because we do not wish to read too much into *t*_0_, we simply treat this parameter as an additive constant.

### Novel features of the Poisson-SLRT model

So far, the present approach differs from previous SLRT-models of choice RT (for a review, see Luce, 1986) with respect to two important features: First, traditional models assume that information is sampled at discrete time points, Δ*t* time units apart, that is, *T*_*j*_ = *j*·Δ*t*, (*j* = 1, 2, …). In the present framework, information is instead sampled approximately θ_*w*_ time units apart when the intensity of the stimulus is weak, and θ_*s*_ time units apart when the intensity is strong. This yields the prediction that more intense targets should on the average be identified faster due to constraints on evidence accumulation imposed by neural waiting times in sensory pathways.

Second, traditional models usually assume that information at each discrete time point is sampled from a stationary distribution, such as the Gaussian (e.g., Ashby, 1983; Stone, 1960; Laming, 1968; Thomas, 1975). According to our model, sampling points instead depend on the IATs between successive neural pulses. These waiting times are governed by the Poisson process and are, therefore, random. The introduction of random sampling times adds an additional element of complexity when seeking an analytical solution for the distribution of first-absorption times for the decision process.^1^

A key strength of the sequential analysis framework in the context of two-choice intensity identification tasks is that if the Poisson approximation of sensory encoding is taken at face value, then the SLRT can be seen to embody the optimal decision rule in this type of task. This is because no other test procedure will yield a smaller expected sample number for neural pulses without also increasing the desired confusion probabilities α and β (Wald and Wolfowitz, 1948). In this regard, the Poisson-SLRT model aligns with the efficient coding hypothesis of Barlow (1961) which posits that sensory systems have evolved to eliminate redundancy in nervous transmission. A significant limitation of choice RT models based on the SLRT, however, is their prediction that correct and incorrect RTs should have identical distributions when conditioned on response class (as discussed by Link and Heath, 1975). Clearly, this prediction is unrealistic for most choice RT tasks, where error RTs tend to be either faster or slower than correct RTs (Maanen et al., 2018). As a result, without further refinement, the Poisson-SLRT model imposes overly rigid constraints on the RT distributions. This shortcoming will be addressed and remedied next.

### Response bias and starting variability

In the context of discrete-time SLRT models, it has been shown that a reasonable account of fast errors necessitates some degree of trial-to-trial variability in the starting point Σ_0_ of the evidence accrual process (Laming, 1968, 1979; Swensson and Green, 1977; Ashby, 1983; Thomas, 1975).^2^ Starting point variability also plays a crucial role in the context of evidence accumulation models more broadly, such as accumulator and drift-diffusion models (e.g., Ratcliff, 2013; Ulrich et al., 2015; Heath, 1981; Brown and Heathcote, 2005). In these models, variability in the starting point can produce quick, erroneous responses, even when overall decision-making ability remains stable.

The present model conceives of the starting point variability as a Gaussian random variable, so that


(9) $$\begin{array}{l} \Sigma_{0}\sim N\left(\mu_{s},\sigma_{s}\right). \end{array}$$

Here, the parameters μ_*s*_ and σ_*s*_ can be read as shorthand for “bias” and “variability,” respectively. The variability parameter, σ_*s*_, reflects trial-to-trial fluctuations in the observer's cognitive state which can impact decision-making consistency. This variability is due to unsystematic influences, such as attention, arousal, or fatigue, that randomly shifts the internal baseline from which the observer starts accumulating information, even if the actual task remains unchanged. It helps explain short error RTs by positing that the observer begins some trials with a pre-activation state that is far removed from baseline.

The bias parameter, μ_*s*_, captures more systematic tendencies in the observer's response strategy, which tilt their choices toward one response option across an entire experiment or set of trials. This idea is somewhat reminiscent of the approach outlined by Ashby (1983) who explored a discrete-time SLRT-model where a constant *k* is added to each increment Λ_*j*_. He demonstrated that this is equivalent to a model in which the absorption barriers drift toward the positive or negative domain, depending on the sign of *k*. In the present model, μ_*s*_ simply captures the idea that the pre-activation state may have a non-zero expected value, in addition to trial-to-trial variability. This implies that, on average, the observer starts each trial with a net bias toward one of the two stimuli. From a functional perspective, μ_*s*_ allows for a better account of the relative latencies between the two types of error RT distributions which would otherwise be too constrained to capture realistic datasets. The merits and drawbacks of these two auxiliary model parameters, response bias (μ_*s*_) and starting point variability (σ_*s*_), will be explored in the discussion following Experiment 1; in particular, in terms of their impact on predicted error rates.

## Experiment 1: Brightness identification

Experiment 1 evaluated how well the Poisson-SLRT model captures performance in a speeded, two-choice brightness identification task. The primary objective was to gauge its predictions against standard behavioral benchmarks, including mean RTs, error rates, and the full distributions of RTs for both correct and incorrect responses. The impact of auxiliary parameters on model fit was assessed by comparing a saturated model (*M*_θ_) to models incorporating only decision bias (*M*_μ_) or starting point variability (*M*_σ_), as well as a reduced model that included neither (*M*_−θ_). For reasons that will become clear once we turn to Experiment 2, a hypersaturated model (*M*_θ+_) which allowed for trial-by-trial variability in transduction rate was also tested. All parameters except for the non-decision time *t*_0_ were allowed to vary freely between stimulus pairings in these five models.

Additionally, we sought to delve deeper into two specific areas of interest: First, we aimed to investigate whether bright visual stimuli are identified faster than dim visual stimuli, aiming to shed light on the role of stimulus intensity on identification latency. The final aim of this experiment was to examine the role of task difficulty in brightness identification. To this end, three levels of stimulus intensity were employed: a dim stimulus, a intermediate stimulus, and a bright stimulus. These stimuli were paired in all possible pairwise combinations, resulting in three experimental conditions: dim vs. intermediate, dim vs. bright, and intermediate vs. bright. The potential effects of stimulus pairing on transduction rates were evaluated by fitting a null model (*M*_0_) which was identical to *M*_−θ_ except rate parameters did not vary between stimulus pairing conditions.

### Method

#### Participants

Thirty participants (20 females, 7 left-handed) with a mean age of 24.2 years (age range: 19–68 years) were recruited from the student pool at the University of Tübingen. One participant failed to complete the entire experiment, yielding a final sample size of *N* = 29. They were offered either a 10€ monetary reward or mandatory course credit as reimbursement for their participation in a single 45-minute session. All participants provided informed consent and reported normal or corrected-to-normal visual acuity. The experiment was approved by the ethics committee for psychological research at the University of Tübingen and was conducted in accordance with the Declaration of Helsinki. Target sample size was determined based on available resources for testing.

#### Apparatus and stimuli

The experiment was controlled by an Esprimo P956/E90+ microcomputer (Fujitsu Limited, Tokyo) running a PsychoPy script (Peirce et al., 2019) on a 64-bit Windows 7 OS. Visual stimuli were presented on a 24.1-inch FlexScan EV2495 LCD monitor (EIZO Corporation, Hakusan) placed approximately 60 cm in front of the viewer. The monitor had a pixel resolution of 1, 900 × 1, 200 and a refresh rate of 60 Hz. Visual stimuli were square patches of light of dim (3 cd/m^2^), intermediate (30 cd/m^2^) or bright (300 cd/m^2^) luminous intensity. The stimuli measured 3 cm width × 3 cm height and were presented in the center of the monitor for a duration of 300 ms. Background illumination was held constant throughout the entire procedure at 0.3 cd/m^2^. The luminous intensity of stimulus materials was measured with a P-9201-TF photometer (Gigahertz Optik, Türkenfeld). Response time was measured from stimulus onset to the registering of the participant's response via a custom set of response keys interfaced via the computer's parallel port. Requested timing specifications were verified through external chronometry using a BlackBox Toolkit (Version 2; Blackbox Toolkit Ltd., York; see Plant, 2016) to ensure that stimulus presentation and response recording was both accurate and consistent. The experiment was conducted in a sound- and light-attenuated booth.

#### Design and procedure

The experiment employed a 2 × 3 factorial design, combining three intensity levels (dim, intermediate, and bright) into three pairwise combinations: (A) dim vs. intermediate, (B) dim vs. bright, and (C) intermediate vs. bright. Each signal pairing constituted a stimulus set administered in separate blocks of trials. Participants completed the entire sequence of blocks twice in a pseudo-random order (e.g., BCA-CAB). Consequently, six blocks of experimental trials were completed within a single session. Within each block, there were 100 trials (50 trials per intensity level). Prior to every experimental block, participants were administered practice blocks comprising 20 trials (10 trials per intensity level) to familiarize themselves with the current stimulus set.

At the beginning of the experimental session, participants were presented with written instructions displayed on the computer monitor. The instructions explained the task's objective (“determine whether the light source is dim or bright”) and emphasized the importance of responding both quickly and accurately. The instructions also displayed the visual stimulus pair relevant to the current set of trials and were repeated before the start of each practice block and experimental block. The stimulus-response mapping for left and right response keys was counterbalanced across participants in ABAB-fashion. Each experimental trial began with the display of a white fixation cross in the center of the screen, measuring 4 mm in diameter and remaining visible for 1 second. Following the fixation period, there was a constant foreperiod of 800 milliseconds (ms) during which the screen remained blank. Subsequently, a stimulus was presented in the center of the screen for 300 ms, followed by a black screen until the participant provided a response. In the event of an incorrect response, the German word for error (“Fehler”) was displayed in a large red font for 500 ms. Participants were given the option to take rests between blocks.

#### Data analysis

The initial step of data analysis involved screening and removing individual outlier RTs shorter than 200 ms or longer than 2,000 ms, which were removed from further analysis. Next, error rates were entered into a 3 × 2 repeated-measures ANOVA to examine the effects of stimulus pairing (3 vs. 30, 30 vs. 300, and 3 vs. 300 cd/m^2^) and stimulus intensity (dim or bright stimulus in the pertinent stimulus set) on accuracy of performance. Similarly, mean RTs for correct responses were also analyzed using a 3 × 2 repeated-measures ANOVA with the factors stimulus pairing and stimulus intensity as categorical predictors.

**Figure 2:**
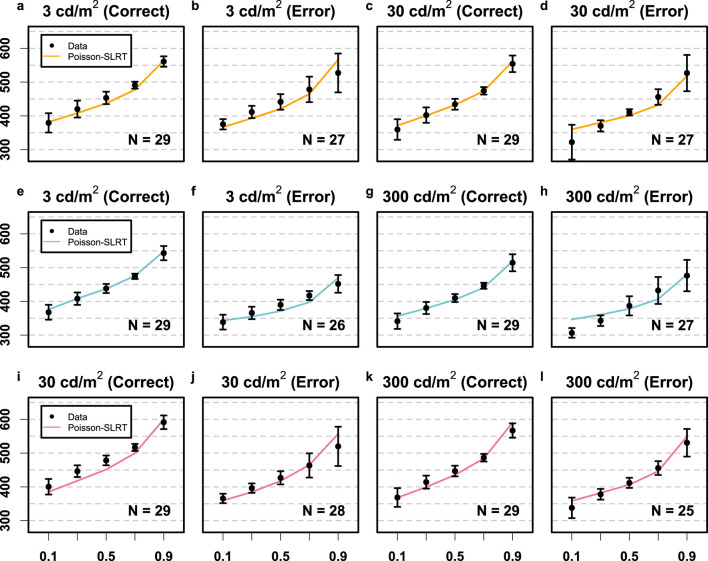
Results Experiment 1 depicted in terms of the fit between empirical data (black circles) and the saturated Poisson-SLRT model (*M*_θ_; colored lines). The orange row **(a–d)** depicts data from the 3 vs. 30 cd/m^2^ condition. The blue **(e–h)** and violet rows **(i–l)** depict data from the 3 vs. 300 and the 30 vs. 300 cd/m^2^ conditions, respectively. The y-axes are RTs in units of ms, and the x-axes are the quantile values of the RT CDFs. Error bars again denote the 95% CIs.

To fit the Poisson-SLRT model variants to the RT data, empirical CDFs were computed for each participant across five quantile values (0.10, 0.30, …, 0.90) for each of 3 × 2 × 2 RT distributions (3 stimulus pairings × 2 stimuli × 2 response categories) using the non-parametric quantile estimator proposed by Harrell and Davis (1982). Subsequently, group-level RT distributions were constructed by averaging quantiles across participants (Ratcliff, 1979). The construction of individual-level CDFs for incorrect RTs was in some cases prohibited because people made very few errors. The number of participants who went into estimating each CDF is denoted in Figure 2. Absorption barriers were calculated for each stimulus pairing following Equations 6, 7.

Next, theoretical quantiles based on 20,000 RTs per stimulus pairing were simulated and fitted iteratively to group-level quantiles using the downhill simplex method (Nelder and Mead, 1965). The fitting routine sought to minimize the χ^2^ discrepancy between observed (empirical) and expected (simulated) quantiles for each of the 12 RT distributions and was repeated for each model listed in Table 1. The best value of the non-decision time *t*_*o*_ was determined for each model separately through a grid search in steps of 10 ms and was always held constant across stimulus pairings. Relative model fit was evaluated using the Bayesian Information Criterion (BIC; Schwarz, 1978). Experimental data and analysis code are available via the Open Science Framework (OSF).^3^

**Table 1:** Comparison of model fits for Experiment 1, tabulated in terms of the number of free parameters (*k*), the chi-square goodness-of-fit statistic (χ^2^), and the Bayesian Information Criterion (BIC).

**Model**	**Adaptation**	**Decision bias**	**Start variability**	**Rate variability**	***k***	**χ^2^**	**BIC**
*M*_0_					3	260	456
*M*_−θ_	✓				7	186	456
*M*_μ_	✓	✓			10	84	423
*M*_σ_	✓		✓		10	108	439
*M*_θ_	✓	✓	✓		13	37	386
*M*_θ+_	✓	✓	✓	✓	16	41	406

Results are shown for the null model (*M*_0_), reduced model (*M*_−θ_), bias model (*M*_μ_), variability model (*M*_σ_), saturated model (*M*_θ_), and hypersaturated model (*M*_θ+_).

### Results

**Figure 3:**
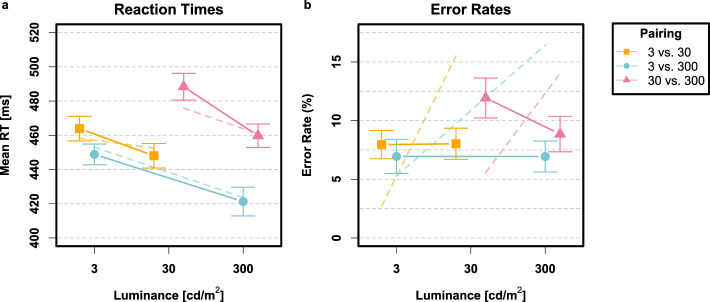
Results from Experiment 1 depicted in terms of mean RTs **(a)** and error rates **(b)** for stimulus pairings 3 vs. 30 (orange squares), 3 vs. 300 (blue circles), and 30 vs. 300 (violet triangles) cd/m^2^, respectively. The dashed lines convey the predictions of the saturated Poisson-SLRT model (*M*_θ_). Error bars signify the within-subjects 95% confidence intervals (95% *CI*s; Loftus and Masson, 1994) with the correction suggested by Morey (2008).

Few responses were too fast (0.25 %) or too slow (0.37 %). The analysis of error rates revealed a main effect of stimulus pairing [*F*_(2, 56)_ = 10.62, *p* < 0.001, $\eta_{G}^{2}=0.051$] and a pairing × intensity interaction [*F*_(2, 56)_ = 6.33, *p* = 0.003, $\eta_{G}^{2}=0.014$] but no main effect of intensity [*F*_(1, 28)_ = 2.04, *p*>0.05]. With respect to RTs, the analysis supported a main effect of stimulus pairing [*F*_(2, 56)_ = 45.38, *p* < 0.001, $\eta_{G}^{2}=0.017$], a main effect of intensity [*F*_(1, 28)_ = 37.48, *p* < 0.001, $\eta_{G}^{2}=0.010$], and a pairing × intensity interaction [*F*_(2, 56)_ = 5.42, *p* = 0.007, $\eta_{G}^{2}=0.0006$]. *Post hoc* contrasts indicated that mean RTs for bright targets (440 ms) were consistently shorter than for dim targets [464 ms; *t*_(28)_ = −6.12, *p* < 0.001, *d*_*z*_ = 1.14], yet bright and dim targets evoked the same proportion of errors [*t*_(28)_ = 1.44, *p*>0.05]. Error rates and mean RTs are depicted together with their within-subjects 95% confidence intervals in Figure 3.

*Poisson-SLRT Model:* The saturated model (*M*_θ_) provided the most parsimonious account of RT CDFs (compare BIC values reported in Table 1). Rate, bias, and variability parameters for the 3 vs. 30 cd/m^2^ pairing condition were ${\hat{\lambda }}_{3}=47$ Hz, ${\hat{\lambda }}_{30}=106$ Hz, ${\hat{\mu }}_{s}=-1.1$, and ${\hat{\sigma }}_{s}=0.75$. For the 3 vs. 300 cd/m^2^ pairing, we obtained ${\hat{\lambda }}_{3}=45$ Hz, ${\hat{\lambda }}_{300}=113$ Hz, ${\hat{\mu }}_{s}=-0.79$, and ${\hat{\sigma }}_{s}=1.29$. Finally, fitting the 30 vs. 300 cd/m^2^ pairing data yielded ${\hat{\lambda }}_{30}=47$ Hz, ${\hat{\lambda }}_{300}=98$ Hz, ${\hat{\mu }}_{s}=-0.63$, and ${\hat{\sigma }}_{s}=0.82$. Non-decision time was ${\hat{t}}_{0}=340$ ms. These 13 free parameters provided an aggregated fit of χ^2^ = 37.0 to the entire set of 60 RT quantiles. Figure 2 illustrates the goodness-of-fit of the saturated model in terms of observed and estimated CDFs for all twelve RT distributions. Visual inspection reveals that much of the discrepancy between theory and data can be attributed to the extreme quantiles of the error RT distributions. The dashed lines in Figure 3 depict predicted mean RTs and error rates. It bears remarking that predicted error rates deviated systematically from their observed values.

### Discussion

Experiment 1 evaluated the Poisson-SLRT model in a two-choice intensity identification task with visual stimuli. First, as predicted, mean RTs were shorter for bright targets compared to dim targets. Notably, this shortening of RTs for bright stimuli could not be explained by a speed-accuracy trade-off, as error rates remained unaffected by visual luminance. The Poisson-SLRT model accounts for a selective effect of stimulus intensity on RTs by proposing that evidence accumulation proceeds more slowly for dim stimuli compared to bright ones due to slower neural transmission rates associated with less intense visual input. This prediction was corroborated by the data, suggesting that shorter RTs observed for bright stimuli have an early sensory origin, whereby shorter IATs for bright stimuli confer a processing advantage already at the earliest stage of the visual processing stream. This finding aligns with prior research by Pins and Bonnet (1996) demonstrating that responses to bright visual stimuli are faster than those to dim stimuli in speeded choice tasks.

Interestingly, achieving an acceptable goodness of fit for all three stimulus pairings necessitated six free rate parameters, despite there being only three visual stimuli. This effectively allowed the neural transmission rates for each target to vary based on the luminance level of its paired stimulus. The need to relax the assumption that transmission rates remain invariant across different pairings may indicate the influence of adaptation processes in the visual pathway, whereby sensory messages are either strengthened or attenuated depending on stimulus pairing. For example, a 30-candela stimulus might be associated with relatively short IATs when paired with a 3 candela stimulus, but with rather long IATs when paired with a 300 candela stimulus. Evidence from both behavioral psychophysics and sensory physiology supports the notion that brightness coding in the visual pathway exhibits these types of contextual dependencies (Stevens and Stevens, 1963; Yeh et al., 1996).

Although the model incorporated pairing-specific transmission rates, the fit between observed and predicted RT CDFs was not perfect. One discrepancy was that the model predicted heavy-tailed RT distributions for all response categories, whereas observed CDFs for incorrect responses sometimes exhibited mild skew. This may be due to the limited data available for estimating the extreme quantiles of incorrect RT distributions, as noisy tail estimates could have influenced the fitting procedure. However, this is not necessarily a flaw. Instead, it indicates that the model is particularly well-suited for accommodating distributions with heavy tails, as typically observed for incorrect RT distributions when more data are available (Rieger and Miller, 2020; Ratcliff, 1979).

A more serious concern arises from the predicted error rates, which systematically deviated from observed error rates. This is clearly shown in the right panel of Figure 3. While the overall predicted error rates for each stimulus pairing were generally close to empirical values, discrepancies emerge when comparing the error rates for individual stimuli. For example, in the 3 vs. 30 cd/m^2^ condition, the average predicted error rate was 9.0%, in close agreement with the observed 8.0%. However, for the dim stimulus, the model predicted a 2.7% error rate, although the observed rate was 8.0%. For the bright stimulus, the observed error rate was again 8.0%, but the predicted rate was notably higher at 15.5%. A similar appeal can be made to the other two conditions. This discrepancy is a direct consequence of the bias parameter, μ_*s*_, which was incorporated to better capture CDFs for incorrect RTs. The negative sign of the μ_*s*_ estimates across all three experimental conditions indicates a consistent bias toward responding ‘dim', which introduced an asymmetry in the decision-making process and skewed predicted error rates for the two visual stimuli. Two potential solutions to this ordeal seem worthwhile exploring: First, incorporating error rates into the fitting procedure's cost function, alongside RT quantiles, could help strike a better balance between fitting RT distributions and error rates. However, how to manage the trade-off between the two does not appear straightforward. Second, allowing the absorption barriers, *A* and *B*, to be free parameters might improve the Poisson-SLRT model's ability to account for both incorrect RT distributions and error proportions at the expense of greatly increased model flexibility.

In interim summary, Experiment 1 assessed whether the Poisson-SLRT model captures human performance in a two-choice brightness identification task. Strong alignment between empirical and theoretical RT CDFs lends some merit to the advanced framework, but incorrect RTs sometimes had a notable impact on fit. Noisy estimates of extreme RT quantiles reflecting distributional tail ends may have contributed to this mismatch. Predicted error rates systematically diverged from observed values due to the model's incorporation of decision bias. This issue could potentially be addressed by factoring error rates into the cost function or by allowing absorption barriers to be free parameters. Empirically, the experiment demonstrated that RTs are shorter for bright stimuli compared to dim stimuli in intensity identification tasks, as predicted by the model.

## Experiment 2: Loudness identification

Experiment 1 aimed to shed new light on the time course of human information processing in an intensity identification task with visual stimuli. However, a fundamental assumption of the proposed model is that the mechanisms underlying intensity identification performance should be modality-general. This follows from the Poisson approximation of intensity coding, which states that all sensory modalities encode the physical intensity of the stimulus monotonically through the firing rates of dedicated single units. It therefore seemed worthwhile to examine whether the mechanisms underlying perceptual decision-making in intensity identification tasks remain invariant for seeing and hearing, since visual and auditory modalities fundamentally convey similar information about stimulus intensity to the brain. Experiment 2 addressed this question by gauging human performance in a two-choice loudness identification task. Notably, neural spike trains in auditory fibers differ from their visual counterparts in that they are phase-locked to the acoustic frequency of the stimulus for sound frequencies below 5 kHz (Pickles, 1988), resulting in an approximately geometric distribution of IATs (Luce, 1993). However, since the geometric distribution is simply the discrete analog of the exponential distribution, it seemed justified to ask whether the proposed model could also effectively account for behavioral loudness identification data.

To foreshadow, a key distinction between Experiments 1 and 2 lay in the nature of the error RTs. In Experiment 1, the brightness identification task produced shorter error RTs than correct responses, whereas in Experiment 2, the loudness identification task yielded slower error RTs. This difference prompted us to reconsider our data-fitting approach for the loudness identification task. Within the broader context of stochastic choice RT models, it is well established that fast errors often necessitate incorporating starting point variability, while slow errors are better captured by introducing variability in the stimulus representation. In the drift-diffusion model, for example, this is typically achieved by allowing the drift rate to fluctuate across trials (Ratcliff, 2013). Adapting this idea within the Poisson-SLRT framework, we drew on an analogous concept from Poisson models of temporal discrimination, which posit that the transduction rate varies across trials (e.g., Ulrich et al., 2022). To formalize this notion, we fitted a hypersaturated model (*M*_θ+_) where trial-wise variability in transduction rate was parameterized as a Gaussian random variable with a mean of 1 and a standard deviation of σ_*r*_.^4^

### Methods

#### Participants

Again, thirty participants (5 males, 3 left-handed) with a mean age of 21.9 years (age range: 19–33 years) were recruited from the same student pool as Experiment 1. They all reported normal hearing and were reimbursed for their participation in a single 45-min session with either a 10€ monetary reward or mandatory course credit. Each participant completed the entire session.

#### Apparatus and stimuli

Auditory stimuli were 220 Hz pure sinusoids presented at three distinct acoustic sound pressure levels: 50, 60, and 70 dB(A). The tones were delivered binaurally through loudspeakers (PowerMax 80/2, TEAC, Tokyo) which flanked the computer monitor at approximately 30 cm distance from the center of the screen. Auditory stimulus amplitudes were calibrated using a CEL-275 sonometer (Casella CEL Instruments Ltd., Hitchin).

#### Procedure

The only procedural difference between Experiments 1 and 2 was the change of sensory modality from vision to audition. Other than this modification, the experiments were conceptually identical.

#### Data analysis

Again, individual outlier responses were addressed by excluding RTs ≤ 200 ms as anticipations and RTs ≥2, 000 ms as misses. Error rates and mean correct RTs were entered into separate 2 × 3 repeated-measures ANOVAs to assess main and interaction effects of stimulus intensity and stimulus pairing. Finally, each model listed in Table 2 was fitted to empirical RT CDFs in a grid search over *t*_0_ using a χ^2^ loss function within the simplex routine. Relative fit was again compared using the BIC. In all respects, the analysis mirrored that of Experiment 1, except we simulated twice as many RTs per condition (40,000) to assure that difficulties encountered fitting error RTs were not artifacts of Monte Carlo noise. Data and analysis code are available as supplementary material online.^5^

**Table 2:** Comparison of model fits for Experiment 2.

**Model**	**Adaptation**	**Decision bias**	**Start variability**	**Rate variability**	***k***	**χ^2^**	**BIC**
*M*_0_					3	443	456
*M*_−θ_	✓				7	417	462
*M*_μ_	✓	✓			10	141	454
*M*_σ_	✓		✓		10	440	463
*M*_θ_	✓	✓	✓		13	151	457
*M*_θ+_	✓	✓	✓	✓	16	86	410

### Results

**Figure 4:**
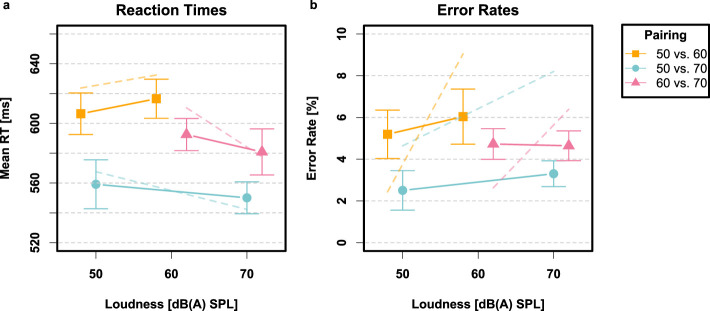
Results from Experiment 2 depicted in terms of mean RTs **(a)** and error rates **(b)** for stimulus pairings 50 vs. 60 (connected orange squares), 50 vs. 70 (connected blue circles), and 60 vs. 70 (connected violet triangles) dB(A) SPL, respectively. Translucent dashed lines conveys the predictions of the hypersaturated model *M*_θ+_. Error bars again signify the 95% CIs.

Very few responses were too fast (0.08%) or too slow (0.1%). The ANOVA for error rates revealed a main effect of stimulus pairing [*F*_(2, 58)_ = 26.45, *p* < 0.001, $\eta_{G}^{2}=0.096$] but no main effect of intensity and no pairing × intensity interaction (all *p*>0.05). With regard to mean RTs, there was also a main effect of stimulus pairing [*F*_(2, 58)_ = 25.24, *p* < 0.001, $\eta_{G}^{2}=0.042$] but again no main effect of intensity (*p* < 0.05). There was, however, an interaction between stimulus pairing and stimulus intensity on mean RTs [*F*_(2, 58)_ = 8.42, *p* < 0.001, $\eta_{G}^{2}=0.002$]. *Post-hoc* contrasts using paired *t*-tests did not support a main effect of intensity within any stimulus pairing conditions, however (all *p*'s >0.05). Error rates and mean RTs are depicted with their associated within-subjects confidence intervals in Figure 4.

**Figure 5:**
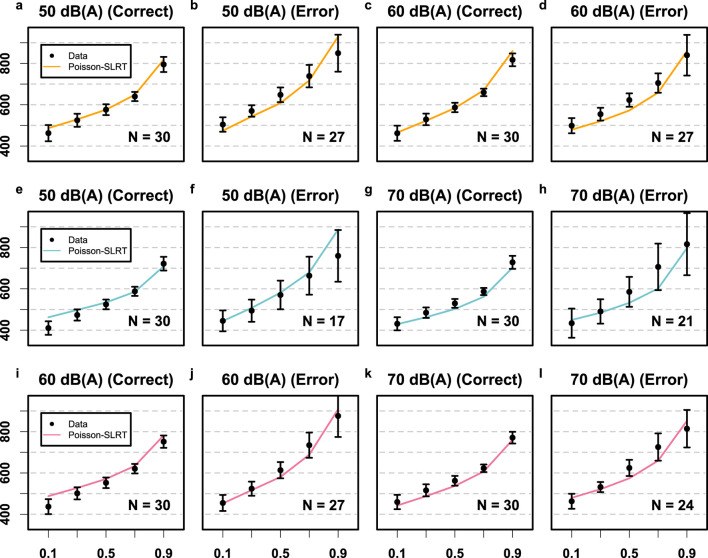
Results Experiment 2 depicted in terms of the fit between empirical data (black circles) and the Poisson-SLRT model (colored lines). The orange row **(a–d)** depicts data from the 50 vs. 60 dB(A) condition. The blue **(e–h)** and violet rows **(i–l)** depict data from the 50 vs. 70 and the 60 vs. 70 dB(A) conditions, respectively. The y-axes are RTs in units of ms, and the x-axes are the quantile values of the RT CDFs. Black error bars again denote the 95% CIs.

*Poisson-SLRT Model:* The hypersaturated model *M*_θ+_ provided the best account of RT CDFs for all three stimulus pairing conditions (compare BIC values listed in Table 2). Estimated rate, bias, and variability parameters for the 50 vs. 70 dB(A) pairing data were ${\hat{\lambda }}_{50}=40$ Hz, ${\hat{\lambda }}_{60}=80$ Hz, ${\hat{\mu }}_{s}=-1.03$, ${\hat{\sigma }}_{s}=0.12$, and ${\hat{\sigma }}_{r}=0.07$. For the 50 vs. 70 dB(A) pairing, their corresponding values were ${\hat{\lambda }}_{50}=43$ Hz, ${\hat{\lambda }}_{70}=104$ Hz, ${\hat{\mu }}_{s}=-1.14$, ${\hat{\sigma }}_{s}=0.80$, and ${\hat{\sigma }}_{r}=0.19$. Finally, fitting the 60 vs. 70 dB(A) pairing data yielded ${\hat{\lambda }}_{50}=40$ Hz, ${\hat{\lambda }}_{70}=85$ Hz, ${\hat{\mu }}_{s}=-0.75$, ${\hat{\sigma }}_{s}=0.14$, and ${\hat{\sigma }}_{r}=0.10$. Non-decision time was ${\hat{t}}_{0}=390$. These 16 free parameters provided an aggregated fit of χ^2^ = 86.4 to the entire set of 60 RT quantiles. The goodness-of-fit for the hypersaturated Poisson-SLRT model is illustrated in Figure 5 in terms of observed and predicted CDFs for all twelve RT distributions. Visual inspection reveals that, again, much discrepancy between model and data can be attributed to the extreme quantiles of the error RT distributions which carried particular leverage. Dashed lines in Figure 3 depict predicted mean RTs and error rates.

### Discussion

Again, it was necessary to allow the rate parameters of the Poisson-SLRT model to vary with the intensity of the paired stimulus in the loudness identification task. This suggests that the adaptation effects on transmission rates observed in Experiment 1 for visual stimuli were similarly present in Experiment 2 for auditory stimuli. More specifically, our model-driven analysis found that the transmission rate for a given stimulus slowed when paired with a relatively loud sound, while it sped up when paired with a softer sound. This pattern indicates that sensory representations of loudness in the auditory pathway are malleable and can be influenced by induced contrast effects.

A difference between Experiments 1 and 2 was the presence of intensity effects on mean RTs in the brightness identification task, which did not emerge in the loudness identification task. It remains unclear why this directional main effect of intensity was absent in the loudness identification task.^6^ One possible explanation lies in the significant interaction between stimulus intensity and stimulus pairing on mean RTs. As shown in Figure 4, this interaction suggests a qualitative crossover effect: RTs were shorter for loud targets in the 50 vs. 70 and 60 vs. 70 dB(A) conditions but longer in the 50 vs. 60 dB(A) condition. Yet, *post-hoc* contrasts of mean RTs within did not statistically confirm this pattern, leaving further interpretation of the interaction inconclusive. The hypersaturated Poisson-SLRT model accommodated the crossover effect on mean RTs, likely through an interaction between estimated decision bias and rate parameters.

Another notable difference was the considerably longer RTs observed in the loudness identification task compared to the brightness identification task. This discrepancy could plausibly be attributed to differences in task difficulty between the two experiments. However, the fact that the estimated residual time *t*_0_ was 50 milliseconds longer in the auditory task challenges a simple task difficulty explanation, because effects of task difficulty would not typically influence residual processes like transmission latency and motor time, which are encompassed in *t*_0_. One speculative hypothesis is that the difference in mean RTs for visual and auditory stimuli may be related to the error rates, which were about half as frequent in the loudness identification task (4.4%) compared to the brightness identification task (8.4%). This suggests that participants might have managed the trade-off between speed and accuracy differently across the two tasks, prioritizing accuracy more in the auditory task and speed more in the visual task.

Errors were generally slower than correct responses in the loudness identification task, whereas the opposite pattern held true for the brightness identification task where errors were faster (compare Figures 3, 4). To account for slow errors, it was necessary to introduce trial-wise variability in the transduction rate within the Poisson-SLRT framework. This adjustment helped explain the ordering of mean RTs for correct vs. incorrect responses in Experiment 2, but resulted in a substantial total of 16 free parameters across all three stimulus pairing conditions. It remains unclear why transduction rate variability was necessary for fitting the auditory data but not the visual. Despite this uncertainty, the model-driven analysis of the data strongly supports this conclusion, even though a clear theoretical explanation is lacking.

Finally, the problematic shifts in predicted error rates for the brightness identification task were similarly reflected in the auditory task, most clearly for the 50 vs. 60 and 60 vs. 70 dB(A) pairings (see Figure 3b). However, a new discrepancy arose when examining the predicted error rates for the 50 vs. 70 dB(A) pairing. Here, the model not only produced asymmetrical shifts around the observed values but also substantially overestimated error rates, increasing the mean from 2.9% to 6.4%. This poor prediction likely stems from an excessively large estimated value of $\hat{\sigma }$, presumably to accommodate the particularly short error RTs in this condition. A straightforward solution would be to impose an upper bound on $\hat{\sigma }$, or alternatively, reduce *t*_0_ across all three conditions to reduce the pull from the shortest correct RT quantiles in panels *e* and *g* of Figure 5. However, as error rates were not formally weighted into our fitting routine we opted against such *post-hoc* adjustments in the present case.

## General discussion

A Poisson model of sensory transduction was fused with a Waldian decision algorithm to account for choice RT and response probability in speeded two-choice intensity identification tasks. Akin to related stochastic evidence accrual models of choice RT, the Poisson-SLRT model necessitated several *post hoc* assumptions about the decision process, such as bias and starting variability, to account for the distribution of error RTs in the brightness identification task (Experiment 1). However, these additional assumptions also introduced undesirable consequences, notably mild asymmetries in error probabilities for dim and bright visual stimuli, despite empirical evidence suggesting these probabilities were equal. Despite this limitation, the model demonstrated admirable fit to the CDFs of RTs for both correct and incorrect responses in Experiment 1. Moreover, as expected, RTs were shorter for bright targets than for dim targets, lending a source of qualitative support for the proposed framework.

Fitting the loudness identification data from Experiment 2 necessitated an additional assumption: variability in transduction rate across trials. Even with this additional free parameter, the model struggled to accommodate the longer RTs observed for error responses compared to correct responses in the auditory task, as reflected in a larger χ^2^ compared to Experiment 1. Additionally, analysis of mean RTs revealed a crossover effect, where responses to loud auditory stimuli did not yield shorter RTs in the 50 vs. 60 dB(A) pairing condition. This crossover effect was qualitatively captured by the hypersaturated Poisson-SLRT model.

Overall, these findings indicate that the Poisson-SLRT model suffers under a curse bestowed upon many stochastic models of choice RT: the need for numerous *post hoc* assumptions in the form of free parameters to accurately capture error RT distributions. This reliance on additional parameters complicates theory testing, as it becomes difficult to ascertain whether a qualitative prediction stems from the model's core assumptions or from the added flexibility provided by *post hoc* modifications. For instance, while main effects of stimulus intensity on mean RTs naturally emerge from the assumption that transduction rates are faster for more intense stimuli, the model's ability to fit error CDFs depended entirely on additional assumptions embodied in μ_*s*_, σ_*s*_, and σ_*r*_.

One straightforward approach to circumvent this issue would be to refrain from fitting error RT distributions altogether since this would eliminate the need for variability and bias in starting point and so forth. However, error RTs provide valuable insights into the mechanisms of perceptual decision-making, making their exclusion disappointing. A more constructive approach would involve addressing the psychological reality of parameters such as starting point bias and variability in research paradigms tailored to selectively influence one of the parameters without affecting the other. For example, manipulating a priori stimulus probabilities could be expected to significantly influence starting point bias while having negligible effects on starting point variability. The effects of implementing reward structures that prioritize accuracy over speed should similarly be captured without altering σ_*s*_. Further research is warranted to properly settle these issues.

A virtue of the Poisson-SLRT model for two-choice intensity identification tasks is its natural extendability to many-choice tasks using the generalization of the SLRT proposed by Sobel and Wald (1949). The Sobel-Wald test replaces the binary random walk with a multidimensional SLRT, where evidence accumulation occurs along multiple decision axes, each corresponding to a pairwise comparison. For a three-choice task, for example, the Sobel-Wald procedure must distinguish among three hypotheses (*H*_1_, *H*_2_, and *H*_3_) requiring two independent comparisons: one between *H*_1_ and *H*_2_, and one between *H*_2_ and *H*_3_. Assuming that the observer waits for each pairwise comparison to terminate before responding, it follows that reaction time should increase as the set size grows, as additional pairwise comparisons are required. Similarly, as the stimulus set size increases, error rates are expected to rise, as each hypothesis in the Sobel-Wald test becomes confusable with a larger number of alternatives.

Another strength of the Poisson-SLRT model becomes evident when compared to the commonly used drift-diffusion model (DDM) of two-choice decision-making tasks. A reviewer noted that under conditions of high input intensities, the SLRT procedure approximates Brownian motion with drift, illustrating a formal connection between the two models. However, a crucial difference lies in their assumptions about the evidence accumulation process. While the DDM allows for relatively flexible drift and diffusion parameters, the Poisson-SLRT model constrains these terms based on its derivation from likelihood ratio computations for exponentially distributed IATs. Furthermore, in the SLRT model, the levels of decision barriers are determined by response errors, whereas in the DDM, these levels remain unconstrained and are treated as free parameters. The constraints of the Poisson-SLRT model, based on an optimal statistical decision framework, enhancing theoretical rigor by limiting the model's ability to accommodate a wide range of empirical patterns. In contrast, DDM's greater flexibility often permits more adaptable data fitting at the expense of weaker theoretical constraints. As philosophers of science have pointed out, well-constrained models tend to contribute more empirical content by generating stronger, more falsifiable predictions (Popper, 1962/2014). Similar sentiments regarding psychological theory testing have been expressed by Roberts and Pashler (2000).

In closing, a brief comment on the possible physiological implementation of the proposed decision-making mechanism seems warranted. Neurons exhibiting non-monotonic tuning curves to stimulus intensity have been identified in both the visual and auditory pathways (e.g., Peirce, 2007; Schreiner and Malone, 2015). These neurons show heightened responses to stimuli near a preferred intensity level, with firing rates diminishing as the discrepancy between preferred value and current input increases. In this regard, they could be viewed as computing likelihood estimates for transmission rates. Consequently, it is conceivable that their activity might contribute to the sequential decision-making process posited by the Poisson-SLRT model. While this connection remains speculative, it represents an promising avenue for future research that could advance our understanding of the neurobiological mechanisms underlying perceptual decision-making in intensity identification tasks.

## Data Availability

The datasets presented in this study can be found in online repositories. The names of the repository/repositories and accession number(s) can be found below: https://osf.io/p4d23/.

## Footnotes

^1^The model also differs from standard random walk models of choice RT based on the Poisson process (e.g., Audley and Pike, 1965; Blurton et al., 2020) where the accumulated difference *D* between two Poisson processes *X*(*t*) and *Y*(*t*) is monitored over time so that *D*(*t*) = *X*(*t*)−*Y*(*t*) is a simple (integer-valued) random walk between two barriers. In these models, the input to the decision process is driven by two simultaneously active channels at each time-point.

^2^But see Rouder (1996) for a demonstration that starting point variability does not always yield fast errors in the discrete-time SLRT-model.

^3^ https://osf.io/8df64/

^4^For completeness, this hypersaturated model was also fit to the data from Experiment 1, but it yielded no meaningful improvements (compare *M*_θ_ and *M*_θ+_ in Table 1). Estimates of σ_*r*_ were so small that transduction rates were essentially always positive.

^5^ https://osf.io/8df64/

^6^Notably, a similar crossover effect of intensity and stimulus modality was reported by Johansson and Ulrich (2025) in a bimodal identification paradigm with redundant auditory-visual stimuli.
